# SAMase of Bacteriophage T3 Inactivates Escherichia coli’s Methionine *S*-Adenosyltransferase by Forming Heteropolymers

**DOI:** 10.1128/mBio.01242-21

**Published:** 2021-08-03

**Authors:** Hadas Simon-Baram, Daniel Kleiner, Fannia Shmulevich, Raz Zarivach, Ran Zalk, Huayuan Tang, Feng Ding, Shimon Bershtein

**Affiliations:** a Department of Life Sciences, Ben-Gurion University of the Negev, Beer-Sheva, Israel; b Macromolecular Crystallography and Cryo-EM Research Center, The National Institute for Biotechnology in the Negev, Ben-Gurion University of the Negev, Beer-Sheva, Israel; c Ilse Katz Institute for Nanoscale Science & Technology, Ben-Gurion University of the Negev, Beer-Sheva, Israel; d Department of Physics and Astronomy, Clemson University, Clemson, South Carolina, USA; University of Washington

**Keywords:** enzyme filamentation, metabolic regulation, virus-host interaction, cryo-EM, molecular dynamics (MD) simulations, bacteriophage T3, SAMase

## Abstract

*S*-Adenosylmethionine lyase (SAMase) of bacteriophage T3 degrades the intracellular SAM pools of the host Escherichia coli cells, thereby inactivating a crucial metabolite involved in a plethora of cellular functions, including DNA methylation. SAMase is the first viral protein expressed upon infection, and its activity prevents methylation of the T3 genome. Maintenance of the phage genome in a fully unmethylated state has a profound effect on the infection strategy. It allows T3 to shift from a lytic infection under normal growth conditions to a transient lysogenic infection under glucose starvation. Using single-particle cryoelectron microscopy (cryo-EM) and biochemical assays, we demonstrate that SAMase performs its function by not only degrading SAM but also by interacting with and efficiently inhibiting the host’s methionine *S*-adenosyltransferase (MAT), the enzyme that produces SAM. Specifically, SAMase triggers open-ended head-to-tail assembly of E. coli MAT into an unusual linear filamentous structure in which adjacent MAT tetramers are joined by two SAMase dimers. Molecular dynamics simulations together with normal mode analyses suggest that the entrapment of MAT tetramers within filaments leads to an allosteric inhibition of MAT activity due to a shift to low-frequency, high-amplitude active-site-deforming modes. The amplification of uncorrelated motions between active-site residues weakens MAT's substrate binding affinity, providing a possible explanation for the observed loss of function. We propose that the dual function of SAMase as an enzyme that degrades SAM and as an inhibitor of MAT activity has emerged to achieve an efficient depletion of the intracellular SAM pools.

## OBSERVATION

*S*-Adenosylmethionine lyase (SAMase) is the first phage-induced protein to appear in T3-infected cells (1). Although it has long been assumed that SAMase uses water molecules to break SAM into methylthioadenosine (MTA) and homoserine, a recent publication has shown that SAMase is, in fact, not a hydrolase but a lyase degrading SAM into MTA and homoserine lactone (2). Degradation of the intracellular SAM pools effectively subdues numerous SAM-utilizing reactions of the host cell, including RNA, DNA, protein, and small-molecule methylation, polyamine synthesis, and production of cofactors (3). Since SAM is an obligatory cofactor required for the action of restriction endonucleases belonging to the type I R-M system (4), it was initially proposed that the primary biological role of SAMase expression was to render T3 bacteriophage immune against host restriction (5). However, this premise was later refuted by showing that phages lacking SAMase activity were nonetheless able to overcome host restriction systems just as efficiently as phages expressing the enzyme (6). Quite unexpectedly, SAMase was found to play a pivotal role in determining the infection strategy of T3 by maintaining its genome in a fully unmethylated state. While T3 is a virulent lytic phage when propagated in rich medium, it initiates a transient lysogenic infection in glucose-starved Escherichia coli cells by blocking its own expression, provided that the infecting T3 DNA is unmethylated (7).

Given the central role played by SAMase in the infection strategy of T3 and the importance of SAM-utilizing reactions to cellular metabolism, it is unsurprising that multiple attempts have been made to purify and characterize this enzyme. It was reported early on that SAMase tightly binds to and copurifies with a host protein factor, yet the identity of the protein or the significance of its interaction with SAMase remained obscure (8, 9).

**FIG 1:**
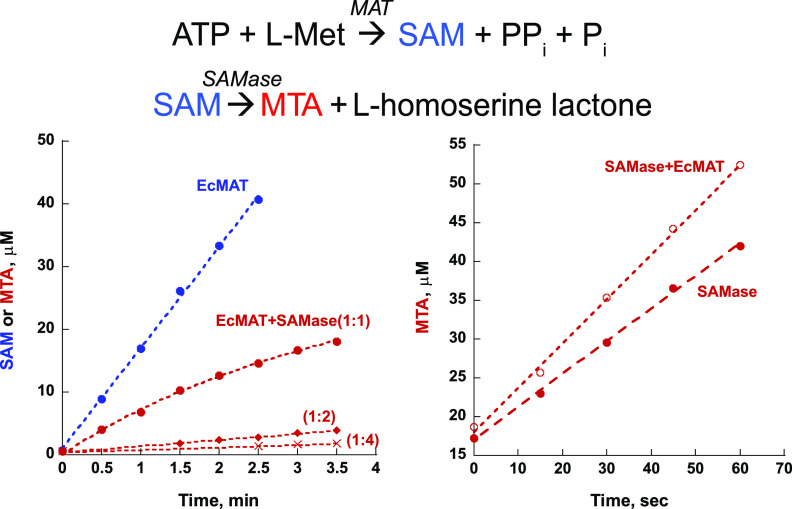
T3 SAMase inhibits E. coli MAT while preserving its own activity. (Left) Synthesis of SAM from ATP and methionine by E. coli MAT was conducted in the absence (blue trace) and presence (red traces) of SAMase. The rate of SAM (blue) and/or methylthioadenosine (MTA) (red) production was monitored by HPLC (see Materials and Methods and Fig. S6D and E). SAMase severely inhibits MAT activity already at the 1:1 EcMAT/SAMase molar ratio; no SAM can be detected, as all SAM molecules produced by the residual MAT activity are immediately degraded into MTA (red traces) and homoserine lactone (not detected). Fourfold molar excess of SAMase almost fully diminishes the MAT activity. (Right) SAMase activity was monitored by following MTA production upon addition of SAM in the absence (filled circles) or presence (empty circles) of E. coli MAT at a 4:1 EcMAT/SAMase molar ratio. Note that no inhibition of SAMase activity is observed. The apparent improvement in the rate of SAM degradation in the presence of MAT might be attributed to the structural stabilization of SAMase.

**SAMase binds to and inhibits MAT.** We began by identifying the unknown host factor that reportedly copurifies with SAMase. To this end, we recombinantly expressed C-terminal His-tagged SAMase in E. coli and performed Ni-NTA affinity purification of the recombinant SAMase, followed by size exclusion chromatography (see Materials and Methods). This purification procedure produced a mix of free dimeric SAMase molecules (∼26 kDa) (see Fig. S1A to D in the supplemental material) and a high-molecular-weight complex comprised of SAMase molecules bound to an unknown host protein that, as previously reported (8, 9), appeared as a single band of ∼43 kDa on SDS-PAGE (Fig. S1E). By applying a shotgun mass spectrometry (MS) analysis and an affinity pulldown assay, we determined that the E. coli protein that binds to and copurifies with SAMase is methionine S-adenosyltransferase (MAT) (see Materials and Methods and Fig. S1A, E, and F). E. coli MAT is a dihedral homotetramer (comprised of dimers of dimers) that catalyzes the condensation of ATP and methionine to produce SAM (10). MAT is an essential enzyme and the only source of SAM in E. coli (11). Thus, SAMase binds to the very enzyme whose unique product it degrades. To understand the functional significance of SAMase-MAT interaction, we measured how the interaction affected the activity of each of the enzymes *in vitro*. Whereas SAMase remained fully active in the presence of MAT, the activity of MAT was severely diminished and, ultimately, blocked with an increase in SAMase concentration (Fig. 1).

10.1128/mBio.01242-21.1 FIG S1 Purification of SAMase under native and denatured conditions. (A) Size-exclusion chromatography (SEC) following Ni-NTA purification of recombinantly expressed 6×His-tagged SAMase under native conditions (see Materials and Methods for details). Two major peaks are marked I and II. (B) Calibration curve for SEC. Molecular weight of protein standards (log scale) is plotted against a ratio of elution volume (Ve) and void volume (Vo). (C) Free SAMase eluted as peak I was concentrated and rerun on the same SEC column. The protein eluted as a single peak at 18.2 ml that, according to molecular weight protein markers (B), corresponds to ∼26 kDa. (D) SDS-PAGE analysis of SAMase cross-linked with 2.5%, 5%, or 10%, vol/vol, glutaraldehyde (GA). Dimeric species are easily distinguished, suggesting that SAMase exists as a dimer in solution (see Materials and Methods for details). (E) Proteins eluted with peaks I and II (A) were collected at several elution aliquots and analyzed in SDS-PAGE. SAMase was found in both peaks. A higher-molecular-weight protein (around ∼43 kDa) was found only in peak II. The protein was excised from the gel (dashed lines) and subjected to MS analysis (see Materials and Methods). (F) Western blot detection with custom raised polyclonal antibodies of E. coli MAT pulled down from a cell lysate with SAMase (see Materials and Methods). Marker is shown on the left. (G) SEC of refolded SAMase purified under denatured conditions (see Materials and Methods). Note the high similarity to SEC separation of natively refolded SAMase (C). (H) Refolded SAMase is an active protein. Michaelis-Menten fit of activity of a refolded SAMase (1 nM) at a range of SAM concentrations (*k*_cat_ = 87.4 s^−1^, *K_m_* = 6 μM) (see Materials and Methods) is shown. (Inset) Michaelis-Menten fit of activity of a natively purified SAMase (3.5 nM) at a range of SAM concentrations (*k*_cat_ = 70 s^−1^, *K_m_* = 6.8 μM). Note the high similarity of obtained activity between refolded and native SAMases. Download FIG S1, PDF file, 0.8 MB. Copyright © 2021 Simon-Baram et al. 2021 Simon-Baram et al. https://creativecommons.org/licenses/by/4.0/ This content is distributed under the terms of the Creative Commons Attribution 4.0 International license.

10.1128/mBio.01242-21.6 FIG S6 SAMase does not inhibit activity of distantly related orthologous MATs. Synthesis of SAM from ATP and methionine of MAT proteins was conducted in the presence of SAMase, and the rate of SAM and/or MTA production was monitored by HPLC. (A and B) Fourfold molar excess of SAMase relative to *N. gonorrhea* MAT (NgMAT) (A) or *U. urealyticum* MAT (UuMAT) (B) did not inhibit MAT activity. (C) Replacement of E. coli MAT (EcMAT) residues interacting with SAMase fully removes activity inhibition. EcMAT residues directly involved in SAMase-MAT interaction were replaced with their homologues in NgMAT (see Fig. S5 for details), and the activity of the resulting mutant E. coli MAT (EcMAT mut) was measured either alone (blue trace, rate of SAM production from ATP and MET) or in the presence of a fourfold molar excess of SAMase (red trace, MTA formation). The apparent increase in UuMAT and EcMAT mutant in the presence of SAMase is best explained by a reduction in product inhibition due to SAM degradation. (D) HPLC traces of 500 μM SAM (orange) and 5’-methylthioadenosine (MTA) (yellow) standards. The standards were prepared in activity buffer and detected at 254 nm. (E) The integrated area of SAM and MTA traces produced by HPLC separation strongly correlates with the injected concentration of standards. Download FIG S6, PDF file, 0.1 MB. Copyright © 2021 Simon-Baram et al. 2021 Simon-Baram et al. https://creativecommons.org/licenses/by/4.0/ This content is distributed under the terms of the Creative Commons Attribution 4.0 International license.

10.1128/mBio.01242-21.5 FIG S5 MAT residues forming interactions with SAMase. (A) E. coli MAT residues forming an interface with a SAMase monomer are shown as orange and blue spheres. The coloring corresponds to the sequence alignment below. Residues in blue are found only in E. coli and other closely related enteric bacteria. The SAMase monomer is shown in green. (B) Sequence alignment of orthologous MATs. Residues involved in SAMase-E. coli MAT interface and their homologs in other MATs are colored orange and blue. Residues replaced in E. coli MAT to break the interaction with SAMase are colored blue. The residues are numbered according to E. coli MAT sequence. Download FIG S5, PDF file, 0.8 MB. Copyright © 2021 Simon-Baram et al. 2021 Simon-Baram et al. https://creativecommons.org/licenses/by/4.0/ This content is distributed under the terms of the Creative Commons Attribution 4.0 International license.

**FIG 2:**
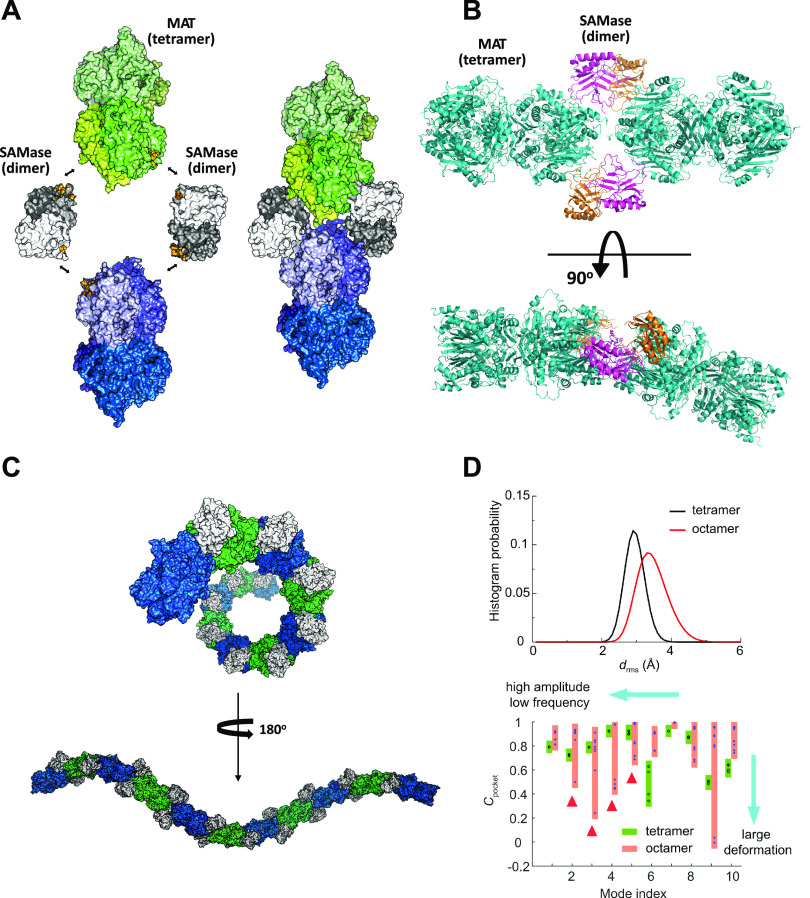
T3 SAMase triggers heteropolymerization and inactivation of E. coli MAT. (A) Two E. coli MAT tetramers (individual monomers are depicted in different shades of green and blue) are brought together by two SAMase dimers (shown in white and gray). The interfaces between the proteins are colored yellow. The properties of the interfaces are summarized in Table S2. Each SAMase monomer interacts with a MAT tetramer and another SAMase monomer. (B) Rotating the SAMase-MAT hetero-oligomer 90° along the axis perpendicular to the dimeric interface of one of the MAT tetramers reveals an ∼30° kink in the assembly of two MAT monomers. MAT tetramers are shown in cyan. SAMase dimers are shown in magenta and orange. (C) The *in silico* reconstructed SAMase-MAT filament reveals helical symmetry (the helix completes a full turn every nine MAT tetramers). MAT tetramers are colored intermittently in green and blue. SAMase dimers are shown in white. See also Fig. S4. (D) DMD simulations and normal mode analyses. (Upper) Root-mean-square deviation, *d*_rms_, of tetramer (black line) and octamer (red line) pocket residues calculated from coarse-grained simulations. Tetramer corresponds to active-site residues in an unbound MAT tetramer. Octamer corresponds to active-site residues within two MAT tetramers bound by two SAMase dimers. (Lower) Mean interresidue dynamic correlation of pocket residues, *C*_pocket_, as a function of normal mode index. The lower the *C*_pocket_ value, the more pocket deformation there is (when *C*_pocket_ = 1, there is no deformation). Four pockets in the tetramer are shown in circles and boxed in green, and eight pockets in the octamer are shown in stars and boxed in orange. Selected correlation matrixes can be found in Fig. S8D. Pocket deformations in the octamer take place at lower-index modes with significantly lower frequencies and higher amplitude (highlighted by red triangles) than the tetramer (see also Fig. S8).

**SAMase-MAT polymerization.** To unravel the structural basis of E. coli MAT inactivation by T3 SAMase, we turned to single-particle cryoelectron microscopy (cryo-EM). To produce sufficient amounts of unbound SAMase, we recombinantly expressed and purified SAMase under denatured conditions and refolded it to a fully active state (see Materials and Methods; see also Fig. S1G and H in the supplemental material). MAT was recombinantly expressed and purified separately (see Materials and Methods). Next, SAMase and MAT were mixed at a 1:1 molar ratio in the presence of SAM and preincubated for 48 h. The mix was then separated by size-exclusion chromatography, and the high-molecular-weight peak comprised of both enzymes was subjected to cryo-EM analysis (see Materials and Methods and Fig. S2A and B). The collected images revealed filamentous structures of various lengths (Fig. S2C). Most two-dimensional (2D) class averages extracted from the images included both MAT and SAMase molecules (Fig. S2D). We solved the structure at 3.6-Å resolution (PDB entry 7OCK; EMDB no. 12809) (Fig. S2E to G and Table S1) and found that the filaments are formed of tetrameric MAT molecules brought together by SAMase dimers (Fig. 2A). Specifically, each SAMase monomer forms an interaction with a MAT monomer and another SAMase monomer, so that each of the two MAT tetramers are joined in a head-to-tail fashion by two SAMase dimers. The interaction between the adjacent MAT molecules is weak, with an interface of only ∼170 Å (see Materials and Methods and Table S2). A SAMase monomer is an α/β protein composed of nine antiparallel β-strands forming a barrel-like structure and two helixes positioned outside the barrel (Fig. 2B and Fig. S3A). The topology of the T3 SAMase fold appears to be unique, even compared to a recently solved phage-encoded Svi3-3 SAMase (2) (PDB entry 6ZMG) (Fig. S3B). Two T3 SAMase monomers interact along ∼1,000-Å^2^ isologous interfaces to form a C2-dimer. The MAT-SAMase interface is smaller and occupies ∼600 to 700Å^2^ (see Materials and Methods and Table S2). The “cracked egg” structure of SAMase dimer pushes one of the two joined MAT tetramers away from the plane (Fig. 2B). This bend between two adjacent MAT tetramers causes a propagation of right-handed helical twists along the entire SAMase-MAT heteropolymer. *In silico* assembly of the SAMase-MAT filamentous structure revealed that SAMase-MAT complex completes a full helical turn every nine MAT tetramers (Fig. 2C and Fig. S4). However, the distribution of MAT tetramers within the individual SAMase-MAT filaments found in cryo-EM images spanned only 2 to 7 tetramers (Fig. S2G), which explains why no helical symmetry could be found in the filamentous structure (see Materials and Methods). Since SAMase-mediated polymerization of MAT proceeds through an open-ended assembly, it is possible that the actual length of the filaments in solution (or *in vivo*) is, in fact, much longer than the distribution of lengths obtained in cryo-EM analysis. Longer filaments probably form entangled structures of reduced solubility. Indeed, a mix of SAMase and MAT proteins subjected directly to cryo-EM analysis without prior separation on a gel filtration column produced clumps of aggregates (not shown).

10.1128/mBio.01242-21.2 FIG S2 Cryo-EM reconstruction of T3 SAMase-E. coli MAT complex. (A) SEC of refolded SAMase and E. coli MAT mix preincubated in the presence of SAM (see Materials and Methods for details). Three major peaks are marked: I (SAMase only), II (MAT only), and III (SAMase+MAT). (B) SDS-PAGE analysis of SAMase-MAT mix. Mix corresponds to a mix of SAMase and MAT prior to SEC. The protein composition of eluted peaks, peak I (SAMase only) and peak III (both SAMase and MAT). Marker is shown on the right. (C) A representative cryo-EM micrograph of SAMase-MAT filaments. (D) Selected 2D class averages. (E) FSC plot of the masked (orange) and unmasked (blue) maps. (F, left) Local resolution map of two MAT tetramers in complex with two SAMase dimers. (Right) Ribbon representation of the structure on the left embedded in the electron density map. The distance between the two most remote points of the opposing eMAT tetramers amounts to 24.9 Å. (G) Distribution of the number of MAT tetramers found in the individual SAMase-MAT filaments. Download FIG S2, PDF file, 2.1 MB. Copyright © 2021 Simon-Baram et al. 2021 Simon-Baram et al. https://creativecommons.org/licenses/by/4.0/ This content is distributed under the terms of the Creative Commons Attribution 4.0 International license.

10.1128/mBio.01242-21.3 FIG S3 Comparison of fold topologies of T3 SAMase and Svi3-3. (A, upper) Ribbon presentation of T3 SAMase monomer. Nine antiparallel β-strands (yellow) assemble into a barrel-like structure and two α-helices (red) are placed outside the barrel. (Lower) Secondary structure elements connected along the primary sequence reveal fold topology of T3 SAMase monomer. Arrows and cylinders represent beta-strands and alpha-helices, respectively. The blue shading depicts beta-pleated sheets. (B, upper) Ribbon presentation of Siv3-3 SAMase monomer (PDB entry 6ZM9). The five antiparallel beta-strands do not form a barrel. (Lower) Fold topology of Siv3-3 SAMase monomer. The lack of the second beta-pleated sheet that closes the barrel-like structure in T3 SAMase is apparent. Download FIG S3, PDF file, 0.4 MB. Copyright © 2021 Simon-Baram et al. 2021 Simon-Baram et al. https://creativecommons.org/licenses/by/4.0/ This content is distributed under the terms of the Creative Commons Attribution 4.0 International license.

10.1128/mBio.01242-21.4 FIG S4 *In silico* reconstruction of SAMase-MAT filament. Filament reconstruction is done by iterative replication and alignment of the SAMase-MAT hetero-oligomeric unit. Download FIG S4, PDF file, 0.3 MB. Copyright © 2021 Simon-Baram et al. 2021 Simon-Baram et al. https://creativecommons.org/licenses/by/4.0/ This content is distributed under the terms of the Creative Commons Attribution 4.0 International license.

10.1128/mBio.01242-21.9 TABLE S1 Data collection and refinement parameters of the cryo-EM reconstruction in this study. Download Table S1, PDF file, 0.1 MB. Copyright © 2021 Simon-Baram et al. 2021 Simon-Baram et al. https://creativecommons.org/licenses/by/4.0/ This content is distributed under the terms of the Creative Commons Attribution 4.0 International license.

10.1128/mBio.01242-21.10 TABLE S2 PISA analysis. Download Table S2, XLSX file, 1.0 MB. Copyright © 2021 Simon-Baram et al. 2021 Simon-Baram et al. https://creativecommons.org/licenses/by/4.0/ This content is distributed under the terms of the Creative Commons Attribution 4.0 International license.

10.1128/mBio.01242-21.8 FIG S8 DMD simulations and normal mode analyses. (A) Structural alignment of a crystal MAT structure (PDB entry 1P7L, blue ribbon) with the cryo-EM MAT structure embedded within MAT-SAMase complex (green ribbon) produced RMSD of 2.16Å. The SAMase dimers are shown as grey ribbons. (B, left) Pocket residues (red) and interface residues (green). The conformation of tetramer is adopted from PDB entry 1P7L. (Right) Pocket residues (upper) and interface residues (bottom). (C) Amplitude and frequency of motions of pocket (active-site) residues as a function of mode index. Tetramer corresponds to active-site residues in an unbound MAT tetramer. Octamer corresponds to active-site residues within two MAT tetramers bound by two SAMase dimers. (D) Correlation matrix of 3rd mode of tetramer pocket (left) and octamer pocket (right). Red regions represent positive correlations with the pairwise residues moving in the same direction, whereas green regions represent negative correlations (anti-correlation) with two residues moving in the opposite direction, which leads to pocket deformation. We can find that anticorrelation motions are rich for octamer but are rare for tetramer, indicating that the octamer pocket is more deformed than the tetramer pocket. The mean interresidue dynamic correlations of pocket residues, *C*_pocket_, are shown on top of each correlation matrix. The variation of *C*_pocket_ as a function of mode index can be found in Fig. 2D. (E, left) Mean interresidue dynamic correlation of interface residues, *C*_interface_, as a function of normal mode index. Lower value of *C*_interface_ means the interface residue motion is larger. The low-frequency modes of tetramer involve large out-of-phase motions of the interface residues, which are inhibited or minimized upon polymerization. (Middle and right) Correlation matrix of 1st mode of tetramer (middle) and octamer (right) interface residues are shown as illustrations. Obvious anticorrelation can be found between 1 to 16 and 17 to 33 interface residues of tetramer, confirming large out-of-phage motions of the 1st normal mode. Collectively, filamentation is found to redistribute the deformation mode of MAT via allosteric effects, shifting top normal modes from large interface deformation and intact pocket of tetramer to restrained interface deformation and distorted pocket of octamer. Download FIG S8, PDF file, 1.9 MB. Copyright © 2021 Simon-Baram et al. 2021 Simon-Baram et al. https://creativecommons.org/licenses/by/4.0/ This content is distributed under the terms of the Creative Commons Attribution 4.0 International license.

**SAMase-MAT interaction is specific.** To validate the functional relevance of the obtained structure, we identified 21 MAT residues participating in the interface formation with SAMase monomers (see Materials and Methods and Table S2). Many of these residues appear to be conserved even among distantly related orthologous MATs (Fig. S5A and B) whose activity we found to be unaffected by the presence of SAMase, such as MATs from Neisseria gonorrhea or Ureaplasma urealyticum (68% and 44% amino acid sequence identity with E. coli MAT, respectively) (Fig. S6A and B). However, 6 out of the 21 interface-forming residues, Asp132, Val 133, Ile 178, Ile 211, Ala 214, and Trp 26, are present only in E. coli or in other closely related enteric bacteria (Fig. S5A and B). We reasoned that the replacement of these residues with their homologs from a SAMase-insensitive MAT should break the MAT-SAMase interaction and, thus, release the inhibition of E. coli MAT activity. To test this assumption, we generated an E. coli-to-*N. gonorrhea* MAT mutant carrying the following substitutions: Asp132Pro, Val133Thr, Ile178Val, Ile211Val, Ala214Pro, and Trp26Leu. As expected, these substitutions have fully removed the inhibitory effect of SAMase (Fig. S6C). Furthermore, dynamic light scattering (DLS) performed on E. coli, *N. gonorrhea*, and mutant E. coli MATs in the absence or presence of SAMase clearly indicated that the introduced substitutions eliminated the complex formation between E. coli MAT and SAMase (Fig. S7A to C). Thus, SAMase-mediated filamentation of E. coli MAT is responsible for its functional inactivation.

10.1128/mBio.01242-21.7 FIG S7 Dynamic light scattering (DLS) analysis of SAMase-MAT complex formation. SAMase, E. coli MAT (EcMAT), E. coli MAT, to *N. gonorrhea* mutant (EcMAT mut), and *N. gonorrhea* MAT (NgMAT) were analyzed alone (blue and red traces) or as SAMase-MAT mix (green traces) after preincubation with SAM (see Materials and Methods for details). (A) A clear shift in the calculated average radius is observed upon mixing of SAMase with EcMAT. (B and C) No such shift can be seen in SAMase-NgMAT (B) or SAMase-EcMAT mut (C) mixes. Download FIG S7, PDF file, 0.1 MB. Copyright © 2021 Simon-Baram et al. 2021 Simon-Baram et al. https://creativecommons.org/licenses/by/4.0/ This content is distributed under the terms of the Creative Commons Attribution 4.0 International license.

**Inhibition of MAT activity is allosteric.** Why does the SAMase-induced polymerization inactivate MAT? Structural alignment of a free MAT homotetramer (PDB entry 1P7L) with the MAT homotetramer bound to SAMase (PDB entry 7OCK) does not explain the causes of MAT inactivation upon filamentation, as both structures almost perfectly overlay (root-mean-square deviation [RMSD] of the atomic positions, 2.16 Å; Fig. S8A). Enzyme catalysis, however, is known to be rooted in the synergy between structure and dynamics (12, 13). Thus, to better understand the mechanism of MAT inhibition, we performed coarse-grained discrete molecular dynamics (DMD) (14) simulations of a MAT tetramer and a MAT octamer joined by two SAMase dimers (see Materials and Methods). We identified MAT pocket (active-site) residues, using 6-Å expansion from the active-site substrates (AMPPNP and methionine in MAT tetramer from PDB entry 1P7L; Fig. S8B). Comparison of RMSD (*d*_rms_) of active-site residues within an unbound MAT tetramer (tetramer pockets) and active-sites residues within two MAT tetramers joined by two SAMase dimers (octamer pockets) calculated from coarse-grained simulations revealed that *d*_rms_ of octamer pocket residues were larger and more broadly distributed than those of the tetramer (Fig. 2D), suggesting that the pocket was less stable and more deformed after filamentation. To determine the mechanism of pocket deformation in the octamer, we next performed normal mode analysis (15). Comparison of top normal modes suggested that the octamer featured lower-frequency and higher-amplitude motions than the tetramer (Fig. S8C). For each normal mode, we defined the overall deformation of MAT pockets by mean interresidue dynamic correlation of pocket residues (*C*_pocket_) and found that the deformation of pockets was significantly larger (low *C*_pocket_ values) in the octamer than the tetramer in low-frequency and large-amplitude modes, which is consistent with our coarse-grained simulations (Fig. 2D and Fig. S8C and D). This extent of active-site deformation potentially weakens MAT affinity to substrates and explains the observed loss of MAT function within filaments. In addition, MAT residues forming the interface with both SAMase and another MAT molecule within a distance cutoff of 0.75 nm in MAT-SAMase-MAT junctions in the high-order complex (Fig. S8B) are likely to have reduced conformational flexibility and more concerted motions than the isolated state. Hence, we employed normal mode analysis to evaluate whether the binding-induced inhibition of modes with out-of-phase motions of the interface residues could effectively amplify the deformation of pockets (Fig. S8E). The motion of interface residues in the isolated MAT tetramer was used as a reference to reveal the effects of filamentation. The deformation of the interface residues was described by mean interresidue dynamic correlation, *C*_interface_. We found that the interface residues in the unbound tetramer undergo high-amplitude out-of-phase motion (low *C*_interface_ values) in the low-frequency modes (Fig. S8E). However, these motions were severely inhibited in the octamer, suggesting that filamentation redistributed the deformation mode by restraining interface motions and amplifying pocket deformation (16). Collectively, DMD simulations together with normal mode analyses suggest that the entrapment of MAT tetramers within the SAMase-mediated filaments leads to an allosteric inhibition of MAT activity via dynamic coupling.

**Conclusions.** Although it is not clear why the methylation state is so important for the behavior of T3 in starved E. coli cells, maintenance of the T3 genome in a fully unmethylated state is known to be the single determining factor in the phage’s ability to establish a transient lysogenic infection upon infection of glucose-starved E. coli cells (7). Considering the importance of the methylation status of the T3 phage genome to its infection strategy, the dual function of SAMase as a SAM-degrading enzyme and as an inhibitor of the only enzyme that produces SAM in the host cell provides a clear evolutionary advantage, as it ensures an efficient prevention of phage genome methylation. To our knowledge, the mechanism by which T3 SAMase inhibits the activity of E. coli MAT, i.e., by mediating the polymerization of MAT tetramers, was not previously reported for any viral protein. Strikingly, however, it resembles the widespread phenomenon of filamentation of central metabolic enzymes in organisms as distinct as bacteria, yeast, worm, drosophila, and human (17). In approximately half of the cases in which the biological role of enzyme filamentation was determined, it was found that filamentation inhibits the enzyme activity, particularly when filamentation was triggered by starvation. In other cases, filamentation led to enzyme activation or even a change in enzyme specificity (17). As our understanding of the regulation of cellular metabolism by means of enzyme filamentation continues to grow, it becomes apparent that the advantage of metabolic filamentation is rooted in its ability to rapidly affect metabolic function in response to environmental transitions without changing enzyme concentration. Our finding that a viral protein can rapidly exert metabolic control over the host cell by recruiting a similar strategy of metabolic filamentation opens an interesting possibility that filamentation of host enzymes is a widespread strategy implemented by viruses to reprogram and subdue the metabolic state of the host.

### SAMase cloning and expression.

A T3 SAMase-encoding gene (UniProt no. P07693) with the addition of six His-encoding codons at the 3′ end was synthesized *de novo* by Integrated DNA Technologies (IDT) and cloned into pZA31 plasmid (Expressys) by Gibson assembly under the P_LTetO-1_ promoter (18). The resulting vector was transformed into a DH5αZ1 E. coli strain, which constitutively expresses the Tet repressor, tightly quenching leaky expression (18). To express the protein, an overnight starter of the strain grown in LB medium at 37°C in the presence of 30 μg/ml chloramphenicol was diluted 1:100 into YT medium supplemented with 30 μg/ml chloramphenicol and grown at 37°C to an optical density (OD) at 600 nm of ∼0.6. At this point, SAMase expression was induced by adding 0.2 μg/ml anhydrotetracycline (aTc). Upon addition of the inducer, the temperature was reduced to 20°C and the cells were allowed to grow overnight. The cells were then harvested by centrifugation (5,000 × *g*, 20 min) and subjected to either native or denatured purification protocols.

### SAMase purification under native conditions.

For purification under native conditions, the harvested cells were preincubated for 30 min on ice with 1 mg/ml lysozyme, sonicated, and centrifuged, and the resulting soluble fraction was applied on a Ni-nitrilotriacetic acid (NTA) column (His Trap FF, 1 ml; GE Healthcare) and purified according to the manufacturer's instructions. The eluted protein was dialyzed at 4°C against buffer A (20% glycerol, 20 mM HEPES, pH 7, 150 mM KCl, and 5 mM dithiothreitol [DTT]), concentrated (Amicon centrifugal filter units, 3-kDa cutoff), and applied on a gel filtration column (Superdex 200 Increase 10/300 GL column; GE Healthcare) preequilibrated with buffer B (25 mM Tris, pH 8.0, 150 mM KCl, 1 mM DTT). SAMase eluted at 18.2 ml, which corresponded to ∼26 kDa (see Fig. S1A to C in the supplemental material). The purified SAMase was dialyzed against buffer A and stored at −20°C in buffer A brought to 50% glycerol.

### SAMase purification under denatured conditions.

Only a small fraction of SAMase purified under native conditions was found in a free unbound form (Fig. S1A). To generate sufficient amounts of free SAMase, we performed purification under denatured conditions followed by refolding. To this end, the harvested cells were lysed in chilled lysis buffer (6 M guanidine hydrochloride, 20 mM sodium phosphate, pH 7.8, 500 mM NaCl) for 1 h and centrifuged at 5,000 × *g*. The lysate supernatant was then loaded on a Ni-NTA column (His Trap FF, 1 ml; GE Healthcare) preequilibrated with a binding buffer (8 M urea, 20 mM sodium phosphate, pH 7.8, 500 mM NaCl). The column was washed with 10 column volumes of the binding buffer. SAMase was eluted with an elution buffer (8 M urea, 20 mM sodium phosphate, pH 4, 500 mM NaCl). The eluted protein sample was diluted to >0.05 mg/ml and dialyzed once against 6 M urea, 20 mM HEPES, pH 7, 150 mM KCl, 5 mM DTT, and 20% glycerol at 4°C. The sample was then dialyzed three more times against buffer A at 4°C. Upon completion of dialysis, the sample was centrifuge at 12,000 × *g* for 30 min. The supernatant was collected and concentrated using a filtration unit (Amicon centrifugal filter units, 3-kDa cutoff). The purified refolded SAMase was stored at −20°C in buffer A brought to 50% glycerol. The refolded SAMase produced an elution pattern on a gel filtration column similar to that of the natively purified protein (elution volume, 18.1 ml), and its activity (*k*_cat_ = 87.4 s^−1^, *K_m_* = 6 μM) was similar to that of the natively purified SAMase (*k*_cat_ = 70 s^−1^, *K_m_* = 6.8 μM) (Fig. S1G to H).

### E. coli MAT cloning and purification.

The gene encoding MAT was amplified directly from the chromosome of E. coli MG1655 using the following primers: forward, CATATGGCAAAACACCTTTTTACGTCCGAGTCCGTCTC; reverse, CTCGAGCTATTAATGGTGATGGTGATGGTGCTTCAGACCGGCAGCATCGCGCAGCAGCTGCGCTTTG. The forward primer introduces the NdeI restriction site. The reverse primer introduces a fused fragment encoding the C-terminal His6× and XhoI restriction site. The gene was cloned into the pET24a expression system using NdeI/XhoI restriction sites and expressed in BL21(DE3) cells. Specifically, an overnight starter was diluted 1:100 and grown at 37°C until the OD at 600 nm was 0.5, after which the expression was induced by the addition of 0.4 mM isopropyl-β-d-thiogalactopyranoside (IPTG) overnight at 30°C. Cells were centrifuged at 4,800 × *g*, and the pellet was stored at −20°C. Cells were lysed by sonication after a 30-min preincubation with 1 mg/ml lysozyme (Sigma) and 500 U Benzonase (Sigma) on ice. The filtered lysate was purified by Ni-NTA on a His-TRAP FF 5-ml column (GE Healthcare) and dialyzed into 25 mM Tris, pH 8.0, 150 mM KCl, 1 mM DTT. This was followed by size-exclusion chromatography (monitored at 280 nm) on a Superdex 200 16/600 column (GE Healthcare) in the same buffer. The eluted protein was concentrated using Amicon centrifugal filters and stored at 4°C. Protein purity was validated by SDS-PAGE analysis.

### Determining the oligomeric status of SAMase in solution.

Based on the primary sequence, the anticipated size of the SAMase monomer is around 17 kDa. The estimation of SAMase size by a gel filtration column using beta amylase (200 kDa), alcohol dehydrogenase (150 kDa), albumin (66 kDa), carbonic anhydrase (29 kDa), and cytochrome *c* (12.4 kDa) as protein standards produced a molecular mass of ∼26 kDa for both natively purified and refolded SAMase (Fig. S1A, B, and G), suggesting that SAMase forms a dimer in solution. To test this possibility, we performed a cross-linking analysis. Specifically, 28 μM SAMase was mixed with glutaraldehyde (G6257; Sigma) to a final concentration of 2.5, 5, or 10%, vol/vol. The protein mixes were then incubated for 30 min at 25°C. Quenching was performed by the addition of one sample volume of 1 M Tris-HCl at pH 8.0 and a half sample volume of 5× concentrated Laemmli sample buffer. Finally, the quenched samples were boiled for 5 min at 60°C and separated by SDS-PAGE. Bands corresponding to dimeric (and, to a lesser extent, tetrameric) species appeared in all chosen glutaraldehyde concentrations, suggesting that SAMase exists primarily as a dimer in solution (Fig. S1D).

### Activity assays.

All enzymatic assays were performed at 37°C in activity buffer (25 mM HEPES, pH 7.5, 100 mM KCl, 10 mM MgCl_2_, 1 mM DTT). The activity of MATs in the absence of SAMase was determined at a saturated concentration of methionine (1 mM), 80 μM ATP, and 250 nM enzyme. The enzymatic reaction was initiated by adding ATP. To measure MAT activity in the presence of SAMase, MATs were preincubated with SAMase at 1:1 to 1:4 molar ratios (250 nM MAT and 250 nM to 1 μM SAMase) for 30 min prior to addition of ATP. To measure the effect of E. coli MAT on SAMase activity, 2 μM SAMase was preincubated for 48 h at 4°C either alone or in the presence of 2 μM E. coli MAT. The preincubated mixes were then diluted 1/40 in the activity buffer, and SAMase activity was measured by adding 80 μM SAM. SAMase catalytic parameters in the absence of MAT were determined at a range of SAM concentrations (0 to 150 μM). SAMase (1 μM) was preincubated for 30 min with 2 μM bovine serum albumin (as a crowder) prior to addition of SAM. Aliquots were removed from the reaction mixes at various time points (up to 4.5 min, every 30 s), and the reactions were stopped by mixing with 10% perchloric acid at a 1:1 ratio. The aliquots were then centrifuged, and the supernatant was separated by high-performance liquid chromatography (HPLC) using a MultiHigh SCX 5-μm, 250- by 4.6-mm column (CS-Chromatographie Service GmbH). The mobile phase consisted of 400 mM ammonium fumarate (adjusted to pH 4.0 using formic acid) at a flow rate of 1 ml/min while measuring absorbance at 254 nm. Data analysis was performed by integrating the peaks corresponding to SAM and methylthioadenosine (MTA) and fitting them to the corresponding calibration curves (Fig. S6D and E). The kinetic constants were derived by fitting the resulting data point to a Michaelis-Menten equation (*V*_max_·[*S*]/*K_m_*+[*S*]).

### MS.

Protein bands of ∼43 kDa were incised from Coomassie-stained SDS-PAGE gel (Fig. S1E) and digested in-gel by trypsin according to the manufacturer's protocol (Promega). Peptides were then extracted and subjected to LC-MS analysis using an Eksigent nano‐HPLC connected to an LTQ Orbitrap XL (Thermo Fisher Scientific). Reverse‐phase chromatography of peptides was performed using an Acclaim PepMap Thermo Fisher scientific (C_18_ column, 15 cm long, 75-μm inner diameter, packed with 300-Å, 5-μm beads). Peptides were separated by a 70‐min linear gradient, starting with 100% buffer A (5% acetonitrile, 0.1% formic acid) and ending with 80% buffer B (80% acetonitrile, 0.1% formic acid) at a flow rate of 300 nl/min. A full scan, acquired at 60,000 resolution, was followed by collision-induced dissociation MS/MS analysis performed for the five most abundant peaks in the data‐dependent mode. Fragmentation (with minimum signal trigger threshold set at 1,000) and detection of fragments were carried out in the linear ion trap. Maximum ion fill time settings were 300 ms for the high‐resolution full scan in the Orbitrap analyzer and 100 ms for MS/MS analysis in the ion trap. E. coli MAT protein was identified and validated using the SEQUEST search algorithms against E. coli (NCBI proteome database collection) operated under the Proteome Discoverer 2.4 software (Thermo Fisher Scientific). Mass tolerance for precursors and fragmentations was set to 10 ppm and 0.8 Da, respectively.

### Affinity pulldown.

E. coli cells were grown in 25 ml LB to an OD at 600 nm of 0.5, harvested by centrifugation (4,500 × *g* for 15 min at 4°C), and lysed with Bug Buster (70921; Novagene) in 50 mM NaH_2_PO_4_, pH 8, 300 mM NaCl, and 250 U of Benzonase nuclease (Millipore) for 30 min at room temperature. The cell lysate was then centrifuged (12,000 × *g* for 10 min at 4°C), and the collected supernatant was mixed with 4 μM 6×His-tagged SAMase. The mix was preincubated for 30 min at room temperature while rotating at 60 rpm. The mix was then applied on Ni-NTA bead spin columns (0.2 ml; 786-943; G BioSciences) preequilibrated with binding buffer (50 mM NaH_2_PO_4_, pH 8, 300 mM NaCl, and 10 mM imidazole). The column was then washed 5× with 2 column volumes of wash buffer (50 mM NaH_2_PO_4_, pH 8, 300 mM NaCl, and 20 mM imidazole), and the protein sample was eluted with 3 column volumes of elution buffer (50 mM NaH_2_PO_4_ pH 8, 300 mM NaCl, and 10 mM imidazole). The eluted sample was separated on SDS-PAGE and analyzed by Western blotting using custom-raised rabbit anti-*E. coli* MAT polyclonal antibodies (Genemed Synthesis Inc.) and goat anti-rabbit horseradish peroxidase (HRP) secondary antibodies (SH026; Abm). HRP on the immunoblot was detected with ECL (Advansta) (Fig. S1F).

### Preparation of protein samples for cryo-EM analysis.

Refolded SAMase (150 μl of 160 μM monomer in buffer A) was mixed with 150 μl of 160 μM (monomer) E. coli MAT in 25 mM Tris, pH 8.0, 1 mM DTT, and 10% glycerol. The two proteins were gently mixed and supplemented with 500 μM SAM. The sample was incubated at room temperature for 1 h and then stored for 48 h at 4°C. Next, 500 μM SAM was added, and the sample was centrifuged at 12,000 × *g* for 20 min at 4°C. The supernatant was then loaded on a size-exclusion column (SEC; Superdex 200 Increase 10/300 GL column; GE Healthcare) preequilibrated with buffer B. High-molecular-weight peaks eluted between the void volume of the SEC (∼8 ml) and 11 ml and comprised both MAT and SAMase (Fig. S2A and B). The collected protein sample was then concentrated using a filtration unit (Amicon ultra centrifugal filters, 3-kDa cutoff) to reach a total protein concentration of 0.2 mg/ml. Cryo-EM samples were prepared by plunge-freezing into liquid ethane; 3 μl of protein solution at a concentration of 0.2 mg/ml was deposited on glow-discharged Quantifoil R 1.2/1.3 holey carbon grids (Quantifoil Micro Tools GmbH, Germany). Samples were manually blotted for 4 s at room temperature and vitrified by rapidly plunging into liquid ethane using a home-built plunging apparatus. The frozen samples were stored in liquid nitrogen until imaging.

### Cryo-EM data acquisition, data refinement, and model building.

Samples were loaded under cryogenic conditions and imaged in low-dose mode on an FEI Tecnai F30 Polara microscope (FEI, Eindhoven, the Netherlands) operated at 300 kV. Data sets were collected using SerialEM (19), using a homemade semiautomated data collection script (20). Images were collected by a K2 Summit direct electron detector fitted behind an energy filter (Gatan Quantum GIF) set to ±10 eV around zero-loss peak. Calibrated pixel size at the sample plane was 1.1 Å. The detector was operated in a dose-fractionated counting mode at a dose rate of ∼8 ē/pixel/second. Each dose-fractionated movie had 50 frames, with a total electron dose of 80 ē/Å^2^. Data were collected at a defocus range of −1.0 to −2.0 μm. Dose-fractionated image stacks were aligned using MotionCorr2 (21), and their defocus values were estimated by Gctf (22). The aligned sum images were used for further processing by cryosparc v2.15.0 (23). Particles were “blob” picked followed by 2D classification. Autopicked particles were based on a template produced from a small subset of manually picked 2D class averages. The picked particles were processed by *ab initio* 2D classification followed by manual inspection and selection of the class averages. Classes were selected based on shape and number of particles. All selected classes had clear signatures of secondary structure elements. Initial 3D reference was prepared from the data set of the untreated sample. Final maps were obtained by 3D refinement with no symmetry imposed (C1). Resolution was assessed by Fourier shell correlation at a 0.143 threshold (Fig. S2E). The final cryo-EM maps following density modification were used for model building. MAT crystal structure (PDB entry 3P7L; resolution, 2.5 Å) was used as an initial template and rigid-body fitted into the cryo-EM density for the highest-resolution state of DNA-PKcs in UCSF Chimera (24) and manually adjusted and rebuilt in Coot (25). Real-space refinement was then performed in Phenix (26). The SAMase structure was built *de novo* into the cryo-EM density map. Following refinement of the highest-resolution state, the MAT-SAMase structure was deposited into the PDB with the codes shown in Table S1.

### Protein interface analysis.

MAT-SAMase and SAMase-SAMase interfaces were characterized using the protein interfaces, surfaces, and assembly (PISA) server (27). The interface area is calculated by PISA as a difference in total accessible surface areas of isolated and interfacing structures divided by two. Change in the solvation free energy upon formation of the interface, Δ*^i^G*, in kcal/mol, is calculated as a difference in total solvation energies of isolated and interfacing structures. Negative Δ*^i^G* corresponds to hydrophobic interfaces and does not include the effect of satisfied hydrogen bonds and salt bridges across the interface. PISA also predicts the formation of hydrogen bonds and salt bridges in the interface. The individual parameters of each interface are summarized in Table S2.

### DLS.

MATs and SAMase were analyzed either separately (18.4 μM MAT and 22 μM SAMase) or together (2.85 μM MAT and 11.4 μM SAMase, 1:4 ratio). All measurements were done in buffer of Tris, pH 8.0, 150 mM KCl, and 1 mM DTT. All samples were supplemented with 500 μM SAM and preincubated for 30 min at room temperature prior to analysis. Spectra were collected in a 1-ml volume by using CGS-3 (ALV, Langen, Germany). The laser power was 20mW at the He-Ne laser line (632.8 nm). Averaged (10 runs) scattered intensities were measured by an ALV/LSE 5004 multiple-tau digital cross correlator, at 60 to 90°, during 30 s at room temperatures. The correlograms were fitted with a version of the program CONTIN (28).

### Coarse-grained DMD simulations.

Coarse-grained simulations were performed with discrete molecular dynamics (DMD) simulations. The details of the DMD algorithm can be found in previous publications (29). To reduce the computational cost, each residue was represented by one Cα bead. The coarse-grained protein model using Cα atoms to represent corresponding residues in both DMD simulations and normal mode analyses is tolerant to potential coordinate errors such as bad contacts or bonds. Residue pairs forming native contact (cutoff of 7.5 Å) experienced attractive *Gō* potentials, and hardcore repulsions were applied to residue pairs without native contacts (30). For each system, 39 independent simulations starting with different initial positions and velocities were performed. Each trajectory lasted 6 million DMD steps, with only the last 4 million simulation data used for equilibrium conformational analysis. The deformation of pocket was characterized by RMSD, $d_{\text{rms}}=\sqrt{\frac{1}{N}\sum_{i\;=\;1}^{n}\sum_{j\;=\;i+1}^{n}{\left(d_{\text{ij}}\;-\;d_{\text{ij}}^{\text{0}}\right)}^{2}}$, where *n* was the number of pocket residues, $N=\frac{n\left(n\;-\;1\right)}{2}$ was the number of pocket residue pairs, and $d_{\text{ij}}^{\text{0}}\;$ and $d_{\text{ij}}$ were the distances between residue *i* and residue *j* in the native state and simulations, respectively.

### Normal mode analyses.

Normal mode analyses were conducted with oGNM server (15). Each residue was represented by one network node with native contacts connected by elastic springs. The residue motions between residue *i* and residue *j* were described by the correlation matrix $c_{\text{ij}}=\frac{u_{i}u_{j}}{\sqrt{u_{i}u_{i}}\;\times \;\sqrt{u_{j}u_{j}}}$, where *u* was the eigenvector of each mode. The amplitude of each mode was $1/\sqrt{\lambda }$, where λ was the eigenvalue of each mode. The overall deformations of pocket and interface residues were characterized by mean interresidue dynamic correlation of residues, i.e., *C*_pocket_ and *C*_interface_.

### Data availability.

The structures were deposited under PDB entry 7OCK and EMDB accession code EMD-12809.

## References

[1] Gefter M, Hausmann R, Gold M, Hurwitz J. 1966. The enzymatic methylation of ribonucleic acid and deoxyribonucleic acid. X. Bacteriophage T3-induced S-adenosylmethionine cleavage. J Biol Chem 241:1995–2006. doi:10.1016/S0021-9258(18)96657-3. 5946625

[2] Guo X, Soderholm A, Kanchugal PS, Isaksen GV, Warsi O, Eckhard U, Triguis S, Gogoll A, Jerlstrom-Hultqvist J, Aqvist J, Andersson DI, Selmer M. 2021. Structure and mechanism of a phage-encoded SAM lyase revises catalytic function of enzyme family. Elife 10:e61818. doi:10.7554/eLife.61818. 33567250 PMC7877911

[3] Fontecave M, Atta M, Mulliez E. 2004. S-adenosylmethionine: nothing goes to waste. Trends Biochem Sci 29:243–249. doi:10.1016/j.tibs.2004.03.007. 15130560

[4] Murray NE. 2000. Type I restriction systems: sophisticated molecular machines (a legacy of Bertani and Weigle). Microbiol Mol Biol Rev 64:412–434. doi:10.1128/MMBR.64.2.412-434.2000. 10839821 PMC98998

[5] Studier FW, Movva NR. 1976. SAMase gene of bacteriophage T3 is responsible for overcoming host restriction. J Virol 19:136–145. doi:10.1128/JVI.19.1.136-145.1976. 781304 PMC354840

[6] Kruger DH, Schroeder C. 1981. Bacteriophage T3 and bacteriophage T7 virus-host cell interactions. Microbiol Rev 45:9–51. doi:10.1128/mr.45.1.9-51.1981. 6261110 PMC281497

[7] Krueger DH, Presber W, Hansen S, Rosenthal HA. 1975. Biological functions of the bacteriophage T3 SAMase gene. J Virol 16:453–455. doi:10.1128/JVI.16.2.453-455.1975. 1097737 PMC354684

[8] Hughes JA, Brown LR, Ferro AJ. 1987. Expression of the cloned coliphage T3 S-adenosylmethionine hydrolase gene inhibits DNA methylation and polyamine biosynthesis in Escherichia coli. J Bacteriol 169:3625–3632. doi:10.1128/jb.169.8.3625-3632.1987. 3301808 PMC212442

[9] Spoerel N, Herrlich P. 1979. Colivirus-T3-coded S-adenosylmethionine hydrolase. Eur J Biochem 95:227–233. doi:10.1111/j.1432-1033.1979.tb12957.x. 110588

[10] Takusagawa F, Kamitori S, Misaki S, Markham GD. 1996. Crystal structure of S-adenosylmethionine synthetase. J Biol Chem 271:136–147. doi:10.1074/jbc.271.1.136. 8550549

[11] Pajares MA, Markham GD. 2011. Methionine adenosyltransferase (s-adenosylmethionine synthetase). Adv Enzymol Relat Areas Mol Biol 78:449–521. doi:10.1002/9781118105771.ch11. 22220481

[12] Eisenmesser EZ, Millet O, Labeikovsky W, Korzhnev DM, Wolf-Watz M, Bosco DA, Skalicky JJ, Kay LE, Kern D. 2005. Intrinsic dynamics of an enzyme underlies catalysis. Nature 438:117–121. doi:10.1038/nature04105. 16267559

[13] Henzler-Wildman KA, Lei M, Thai V, Kerns SJ, Karplus M, Kern D. 2007. A hierarchy of timescales in protein dynamics is linked to enzyme catalysis. Nature 450:913–916. doi:10.1038/nature06407. 18026087

[14] Ding F, Dokholyan NV. 2005. Simple but predictive protein models. Trends Biotechnol 23:450–455. doi:10.1016/j.tibtech.2005.07.001. 16038997

[15] Yang LW, Rader AJ, Liu X, Jursa CJ, Chen SC, Karimi HA, Bahar I. 2006. oGNM: online computation of structural dynamics using the Gaussian Network Model. Nucleic Acids Res 34:W24–W31. doi:10.1093/nar/gkl084. 16845002 PMC1538811

[16] Vishweshwaraiah YL, Chen J, Dokholyan NV. 2021. Engineering an allosteric control of protein function. J Phys Chem B 125:1806–1814. doi:10.1021/acs.jpcb.0c11640. 33566608 PMC10658643

[17] Park CK, Horton NC. 2019. Structures, functions, and mechanisms of filament forming enzymes: a renaissance of enzyme filamentation. Biophys Rev 11:927–994. doi:10.1007/s12551-019-00602-6. 31734826 PMC6874960

[18] Lutz R, Bujard H. 1997. Independent and tight regulation of transcriptional units in Escherichia coli via the LacR/O, the TetR/O and AraC/I1-I2 regulatory elements. Nucleic Acids Res 25:1203–1210. doi:10.1093/nar/25.6.1203. 9092630 PMC146584

[19] Mastronarde DN. 2005. Automated electron microscope tomography using robust prediction of specimen movements. J Struct Biol 152:36–51. doi:10.1016/j.jsb.2005.07.007. 16182563

[20] Davidov G, Abelya G, Zalk R, Izbicki B, Shaibi S, Spektor L, Shagidov D, Meyron-Holtz EG, Zarivach R, Frank GA. 2020. Folding of an intrinsically disordered iron-binding peptide in response to sedimentation revealed by cryo-EM. J Am Chem Soc 142:19551–19557. doi:10.1021/jacs.0c07565. 33166133 PMC7677926

[21] Zheng SQ, Palovcak E, Armache JP, Verba KA, Cheng Y, Agard DA. 2017. MotionCor2: anisotropic correction of beam-induced motion for improved cryo-electron microscopy. Nat Methods 14:331–332. doi:10.1038/nmeth.4193. 28250466 PMC5494038

[22] Zhang K. 2016. Gctf: real-time CTF determination and correction. J Struct Biol 193:1–12. doi:10.1016/j.jsb.2015.11.003. 26592709 PMC4711343

[23] Punjani A, Rubinstein JL, Fleet DJ, Brubaker MA. 2017. cryoSPARC: algorithms for rapid unsupervised cryo-EM structure determination. Nat Methods 14:290–296. doi:10.1038/nmeth.4169. 28165473

[24] Pettersen EF, Goddard TD, Huang CC, Couch GS, Greenblatt DM, Meng EC, Ferrin TE. 2004. UCSF Chimera–a visualization system for exploratory research and analysis. J Comput Chem 25:1605–1612. doi:10.1002/jcc.20084. 15264254

[25] Emsley P, Lohkamp B, Scott WG, Cowtan K. 2010. Features and development of Coot. Acta Crystallogr D Biol Crystallogr 66:486–501. doi:10.1107/S0907444910007493. 20383002 PMC2852313

[26] Afonine PV, Poon BK, Read RJ, Sobolev OV, Terwilliger TC, Urzhumtsev A, Adams PD. 2018. Real-space refinement in PHENIX for cryo-EM and crystallography. Acta Crystallogr D Struct Biol 74:531–544. doi:10.1107/S2059798318006551. 29872004 PMC6096492

[27] Krissinel E, Henrick K. 2007. Inference of macromolecular assemblies from crystalline state. J Mol Biol 372:774–797. doi:10.1016/j.jmb.2007.05.022. 17681537

[28] Provencher SW. 1982. Contin–a general-purpose constrained regularization program for inverting noisy linear algebraic and integral-equations. Comp Phys Commun 27:229–242. doi:10.1016/0010-4655(82)90174-6.

[29] Proctor EA, Ding F, Dokholyan NV. 2011. Discrete molecular dynamics. WIREs Comput Mol Sci 1:80–92. doi:10.1002/wcms.4.

[30] Ding F, Dokholyan NV, Buldyrev SV, Stanley HE, Shakhnovich EI. 2002. Molecular dynamics simulation of the SH3 domain aggregation suggests a generic amyloidogenesis mechanism. J Mol Biol 324:851–857. doi:10.1016/s0022-2836(02)01112-9. 12460582

