# Supplementary figures and images for: SAMase of Bacteriophage T3 Inactivates Escherichia coli’s Methionine S-Adenosyltransferase by Forming Heteropolymers
Source: mBio. 2021 Aug 3;12(4):e01242-21. doi: 10.1128/mBio.01242-21 (PMC8406200; doi:10.1128/mBio.01242-21)

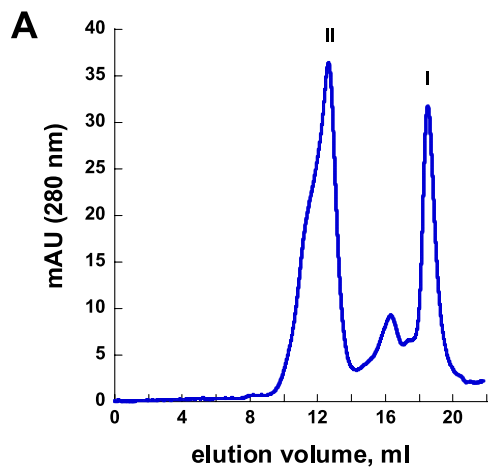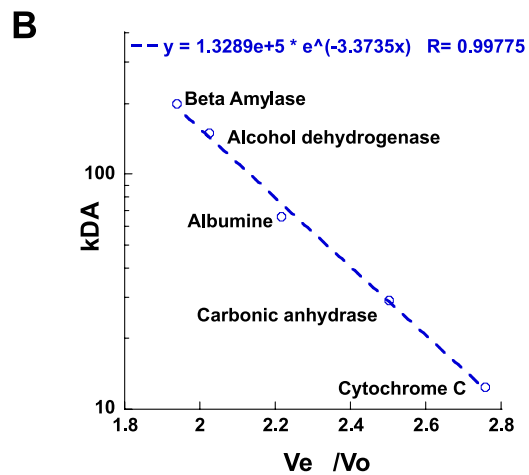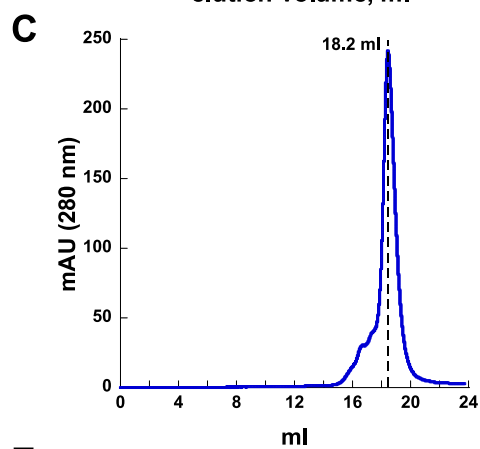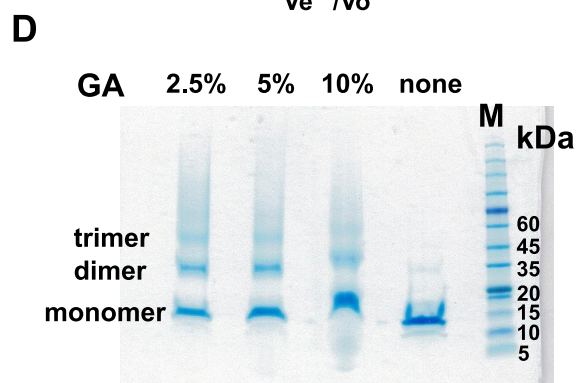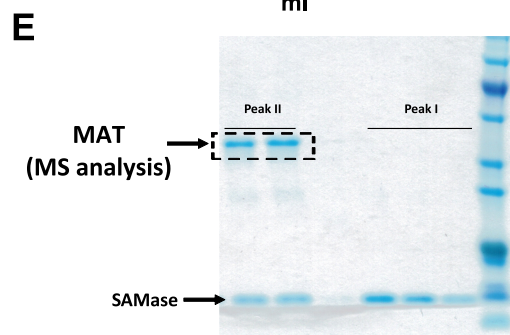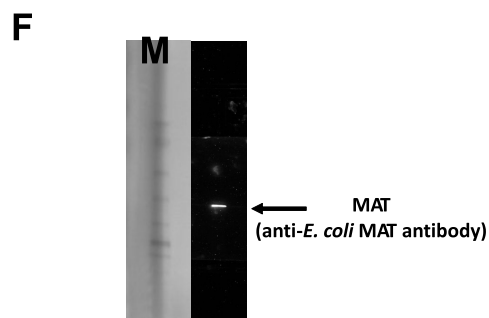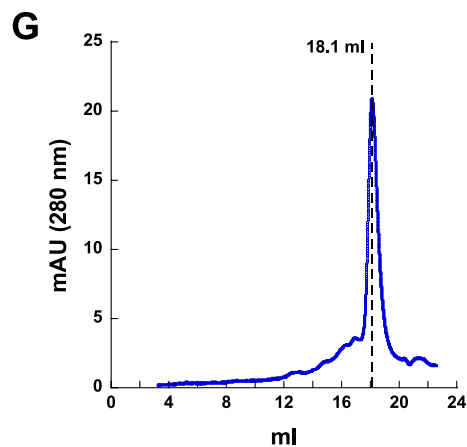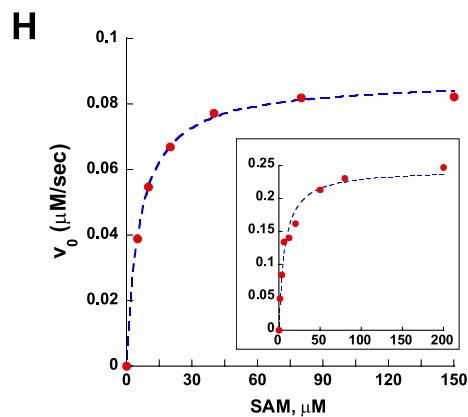

Supplement: FIG S1 [file mbio.01242-21-sf001.pdf]

**A**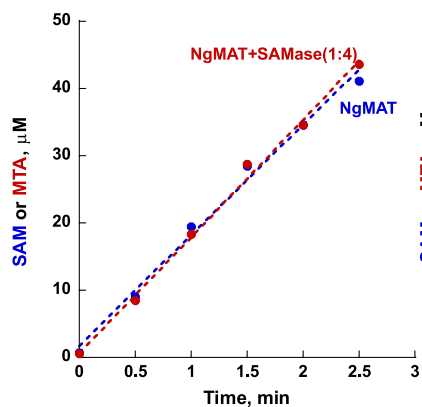**B**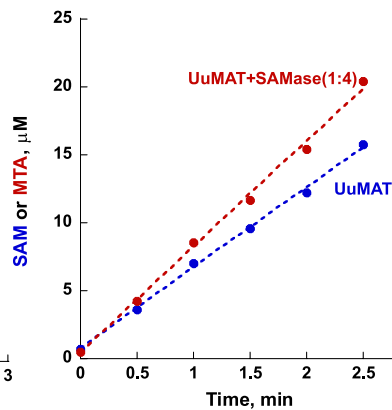**C**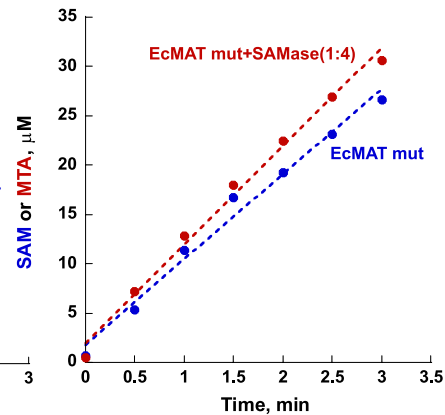**D**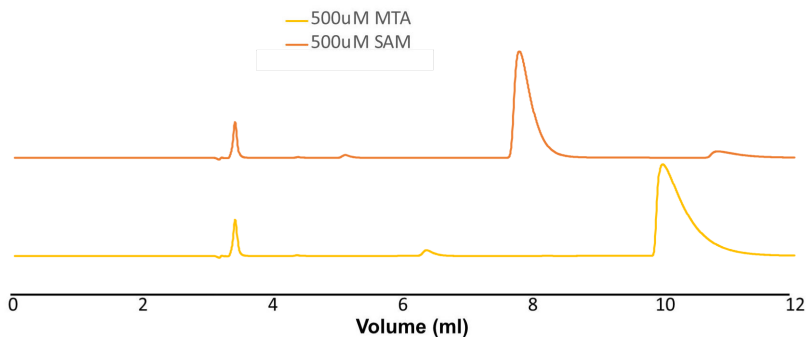**E**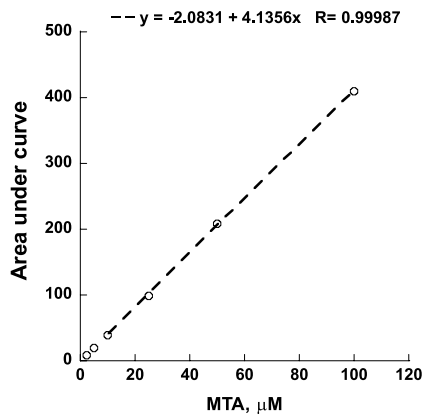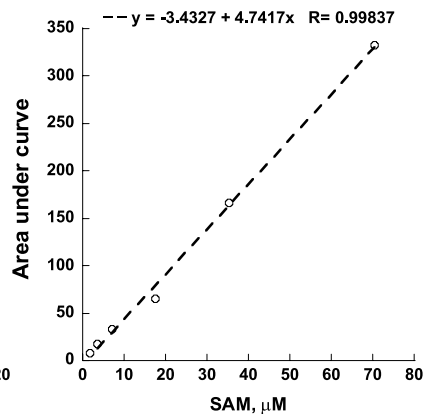

Supplement: FIG S6 [file mbio.01242-21-sf006.pdf]

**A**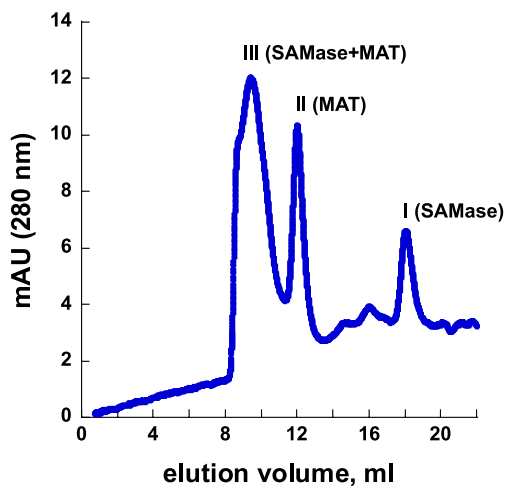**B**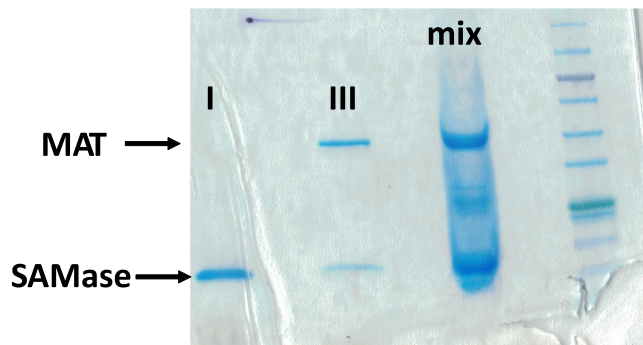**C**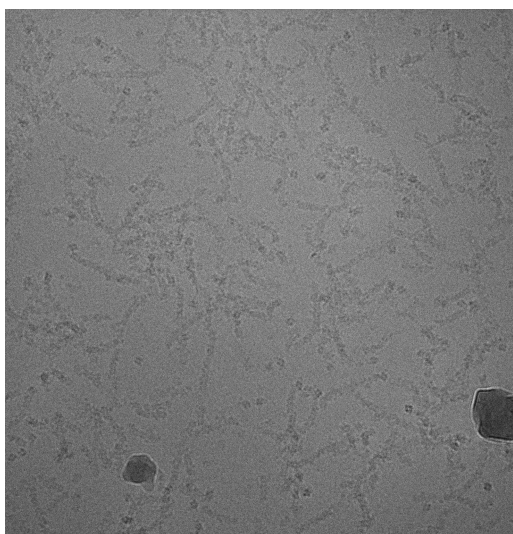**D**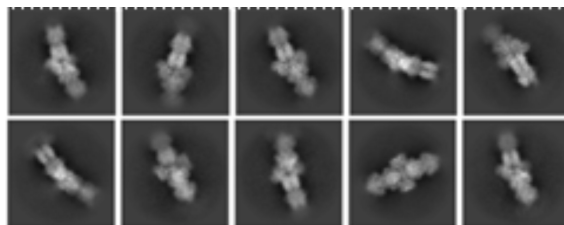**E**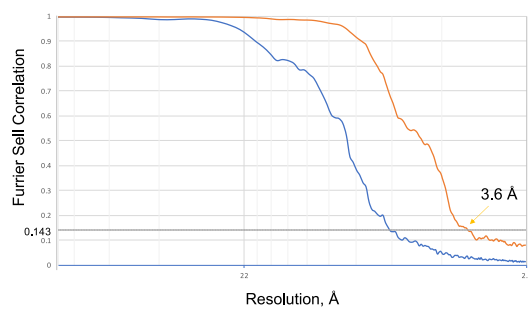**F**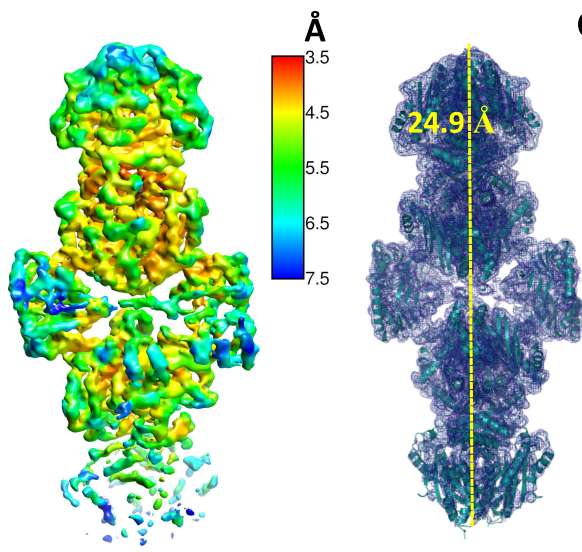**G**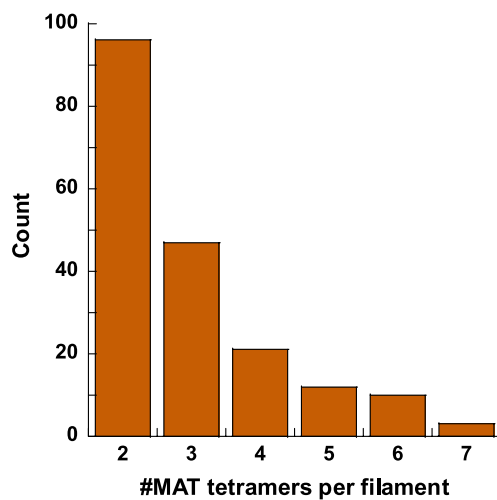

Supplement: FIG S2 [file mbio.01242-21-sf002.pdf]

**A****T3 SAMase**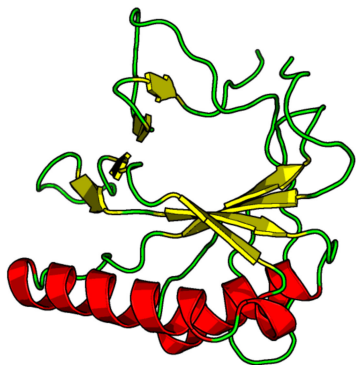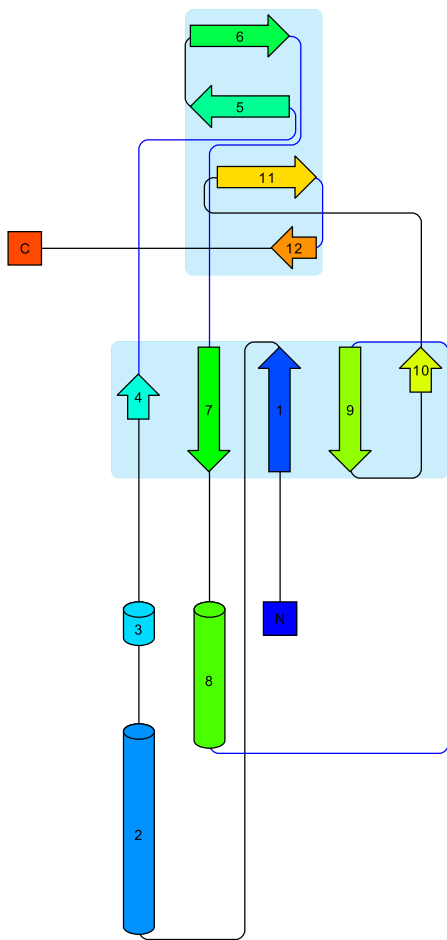**B****Svi3-3 SAMase**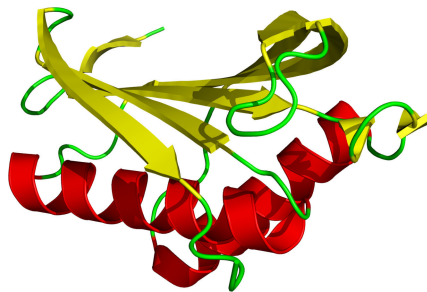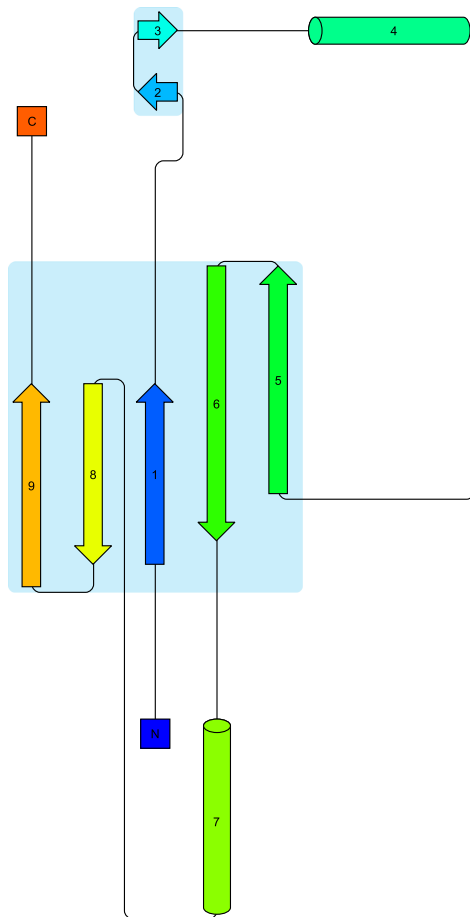

Supplement: FIG S3 [file mbio.01242-21-sf003.pdf]

**A**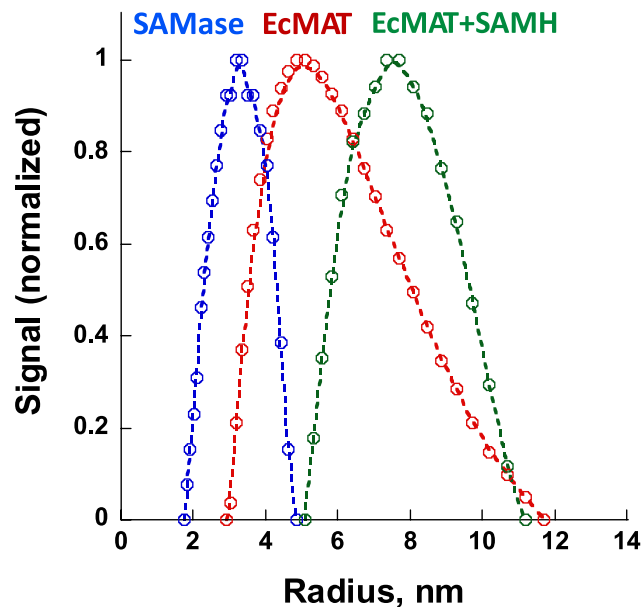**B**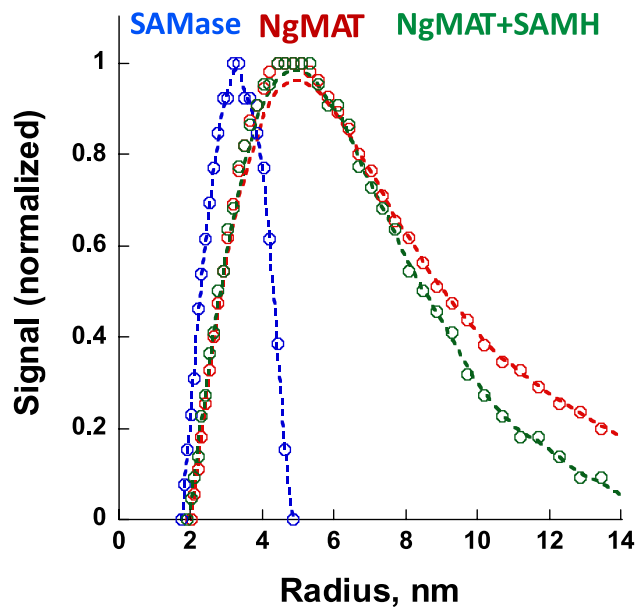**C**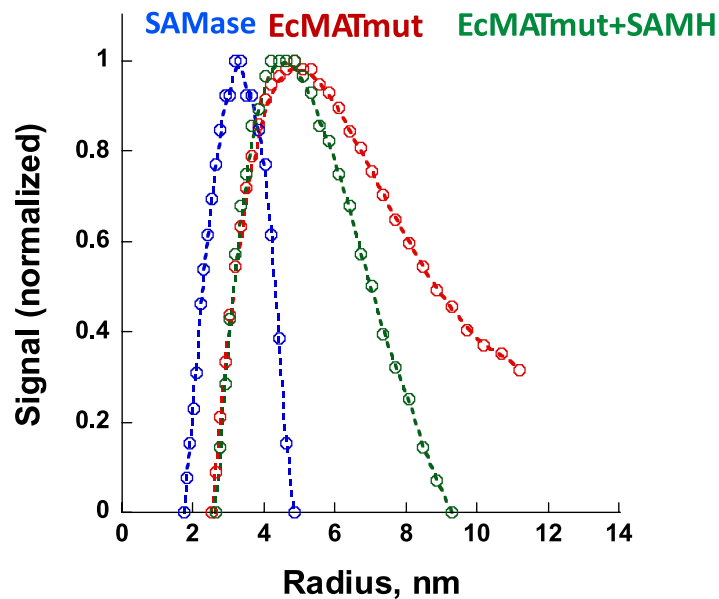

Supplement: FIG S7 [file mbio.01242-21-sf007.pdf]
