# Supplementary material for: SAMase of Bacteriophage T3 Inactivates Escherichia coli’s Methionine S-Adenosyltransferase by Forming Heteropolymers
Source: mBio. 2021 Aug 3;12(4):e01242-21. doi: 10.1128/mBio.01242-21 (PMC8406200; doi:10.1128/mBio.01242-21)
Supplement: FIG S5 [file mbio.01242-21-sf005.pdf]

A

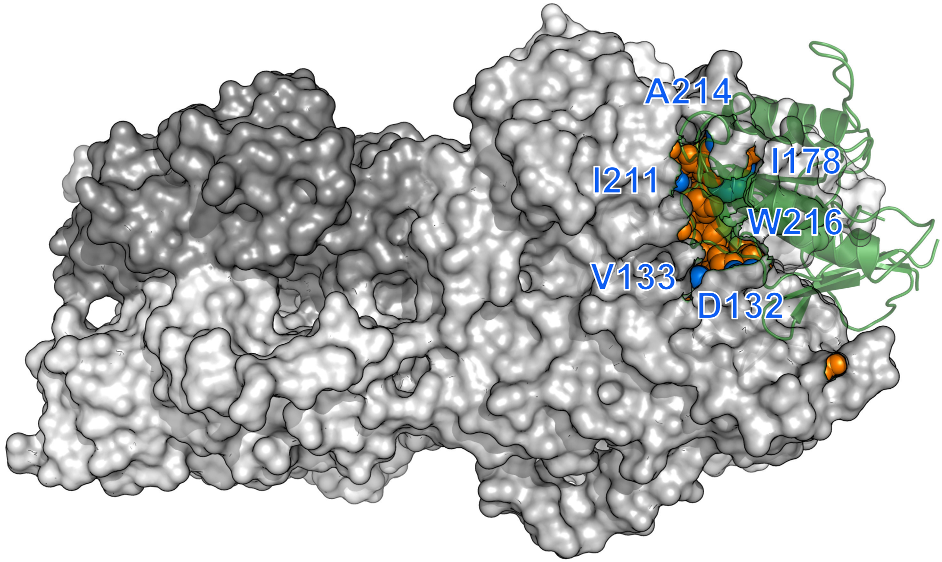

B

```
Ureaplasma_urealyticum   -------------MQYKKIITSESVGAGHPDKICDQISDAILDECLSQDQNSRVACEVLACNRLIVIAGEITTHAYVDVVKTAWEIIKPLGYDEND-------F
Homo_sapiens_MAT1        MNGPVDGLCDHSLSEGVFMFTSESVGEGHPDKICDQISDAVLDAHLKQDPNAKVACETVCKTGMVLLCGEITSMAMVDYQRVVRDTIKHIGYDDSAKGFDFKTC
Homo_sapiens_MAT2        MNGQLNGFHEAFIEEGTFLFTSESVGEGHPDKICDQISDAVLDAHLQQDPDAKVACETVAKTGMILLAGEITSRAAVDYQKVVREAVKHIGYDDSSKGFDYKTC
Bacillus_subtilis        ------------MSKNRRLFTSESVTEGHPDKICDQISDSILDEILKKDPNARVACETSVTTGLVLVSGEITTSTYVDIPKTVRQTIKEIGYTRAKYGFDAETC
Neisseria_gonorrhoeae    --------------MSEYLFTSESVSEGHPDKVADQVSDAILDAILAQDPKARVAAETLVNTGLCVLAGEITTTAQVDYIKVARETIKRIGYNSSELGFDANGC
Salmonella_typhimurium   --------------MAKHLFTSESVSEGHPDKIADQISDAVLDAILQQDPKARVACETYVKTGMVLVGGEITTSAWVDIEEITRNTVREIGYVHSDMGFDANSC
Escherichia_coli         --------------MAKHLFTSESVSEGHPDKIADQISDAVLDAILEQDPKARVACETYVKTGMVLVGGEITTSAWVDIEEITRNTVREIGYVHSDMGFDANSC
Klebsiella_pneumoniae    --------------MAKHLFTSESVSEGHPDKIADQISDAVLDAILEQDPKARVACETYVKTGMVLVGGEITTSAWVDIEEITRNTVREIGYVHSDMGFDANSC

                         132-133   178
Ureaplasma_urealyticum   TIISNVNKQSVDIAQSVDKTN-----------KNLIGAGDQGIVFGYACDETPQYMPLTSVLAHELLKEIERQRRSKEFIKIQADMKSQVSIDYSNST-----P
Homo_sapiens_MAT1        NVLVALEQQSPDIAQCVHLDR----------NEEDVGAGDQGLMFGYATDETEESMPLTIILAHKLNARMADLRRSGLLPWLRPDSKTQVTVQYMQDNGAVIPV
Homo_sapiens_MAT2        NVLVALEQQSPDIAQGVHLDR----------NEEDIGAGDQGLMFGYATDETEECMPLTIVLAHKLNAKLAELRRNGTLPWLRPDSKTQVTVQYMQDRGAVLPI
Bacillus_subtilis        AVLTSIDEQSADIAMGVDQALEAREGTMSDEEIEAIGAGDQGLMFGYACNETKELMPLPISLAHKLARRLSEVRKEDILPYLRPDGKTQVTVEYDE-NNK--PV
Neisseria_gonorrhoeae    AVGVYYDQQSPDIAQGVNEGEGI---------DLNQGAGDQGLMFGYACDETPTLMPFAIYYSHRLMQRQSELRKDGRLPWLRPDAKAQLTVVYDSETGK--VK
Salmonella_typhimurium   AVLSAIGKQSPDINQGVDRAD-----------PLEQGAGDQGLMFGYATNETDVLMPAPITYAHRLVQRQAEVRKNGTLPWLRPDAKSQVTFQYD--DGK--IV
Escherichia_coli         AVLSAIGKQSPDINQGVDRAD-----------PLEQGAGDQGLMFGYATNETDVLMPAPITYAHRLVQRQAEVRKNGTLPWLRPDAKSQVTFQYD--DGK--IV
Klebsiella_pneumoniae    AVLSAIGKQSPDINQGVDRAD-----------PLEQGAGDQGLMFGYATNETDVLMPAPVTYAHRLVQRQAEVRKNGTLPWLRPDAKSQVTFQYD--DGK--IV

                         211 214,216
Ureaplasma_urealyticum   LIETMLVSIQHDEDYDVEYFNKKVSA-IMEQIAKKYNLNTNFKKIINSSGRFVIGGPIGDTGLTGRKIIVDTYGGVGHHGGGAFSGKDPTKVDRSASYFARWIA
Homo_sapiens_MAT1        RIHTIVISVQHNEDITLEEMRRALKEQVIRAVVPAKYLDEDTVYHLQPSGRFVIGGPQGDAGVTGRKIIVDTYGGWGAHGGGAFSGKDYTKVDRSAAYAARWVA
Homo_sapiens_MAT2        RVHTIVISVQHDEEVCLDEMRDALKEKVIKAVVPAKYLDEDTIYHLQPSGRFVIGGPQGDAGLTGRKIIVDTYGGWGAHGGGAFSGKDYTKVDRSAAYAARWVA
Bacillus_subtilis        RIDAIVISTQHHPEITLEQIQRNIKEHVINPVVPEELIDEETKYFINPTGRFVIGGPQGDAGLTGRKIIVDTYGGYARHGGGAFSGKDATKVDRSAAYAARYVA
Neisseria_gonorrhoeae    RIDTVVLSTQHDPAISHEELSKAVIEQIIKPVLPPELLTDETKYLINPTGRFVIGGPQGDCGLTGRKIIVDTYGGAAPHGGGAFSGKDPSKVDRSAAYACRYVA
Salmonella_typhimurium   GIDAVVLSTQHAEDIDQKSLQEAVMEEIIKPILPSEWLNTSTKFFINPTGRFVIGGPMGDCGLTGRKIIVDTYGGMARHGGGAFSGKDPSKVDRSAAYAARYVA
Escherichia_coli         GIDAVVLSTQHSEEIDQKSLQEAVMEEIIKPILPAEWLTSATKFFINPTGRFVIGGPMGDCGLTGRKIIVDTYGGMARHGGGAFSGKDPSKVDRSAAYAARYVA
Klebsiella_pneumoniae    GIDAVVLSTQHAEDIDQKSLQEAVMEEIIKPILPTEWLNASTKFFINPTGRFVIGGPMGDCGLTGRKIIVDTYGGMARHGGGAFSGKDPSKVDRSAAYAARYVA

Ureaplasma_urealyticum   KNVVAAKLAKQCEIQLAFAIGQPQPVAMYVNTFNTNLIDETKIFEAIKKSFNFDIKTFINDLNLWTTKYLPVATYGHFGRDDLDLSWEKLNKVEDLIKNSK--
Homo_sapiens_MAT1        KSLVKAGLCRRVLVQVSYAIGVAEPLSISIFTYGTSQKTERELLDVVHKNFDLRPGVIVRDLDLKKPIYQKTACYGHFGRS--EFPWEVPRKLVF--------
Homo_sapiens_MAT2        KSLVKGGLCRRVLVQVSYAIGVSHPLSISIFHYGTSQKSERELLEIVKKNFDLRPGVIVRDLDLKKPIYQRTAAYGHFGRD--SFPWEVPKKLKY--------
Bacillus_subtilis        KNIVAAELADSCEVQLAYAIGVAQPVSISINTFGSGKASEEKLIEVVRNNFDLRPAGIIKMLDLRRPIYKQTAAYGHFGRHDVDLPWERTDKAEQLRKEALGE
Neisseria_gonorrhoeae    KNIVAAGLATQCQIQVSYAIGVAEPTSISIDTFGTGKISEEKLIALVCEHFDLRPKGIVQMLDLLRPIYGKSAAYGHFGREEPEFTWERTDKAASLKAAAGL-
Salmonella_typhimurium   KNIVAAGLADRCEIQVSYAIGVAEPTSIMVETFGTEKVPAEQLILLVREFFDLRPYGLIQMLDLLHPIYKETAAYGHFGRE--NFPWEKTDKAQLLRDAAGLK
Escherichia_coli         KNIVAAGLADRCEIQVSYAIGVAEPTSIMVETFGTEKVPSEQLTLLVREFFDLRPYGLIQMLDLLHPIYKETAAYGHFGRE--HFPWEKTDKAQLLRDAAGLK
Klebsiella_pneumoniae    KNIVAAGLADRCEIQVSYAIGVAEPTSIMVETFGTEKVPSEQLTLLVREFFDLRPYGLIQMLDLLHPIYKETAAYGHFGRE--HFPWEKTDKAALLREAAGLK
```
