# Supplementary material for: SAMase of Bacteriophage T3 Inactivates Escherichia coli’s Methionine S-Adenosyltransferase by Forming Heteropolymers
Source: mBio. 2021 Aug 3;12(4):e01242-21. doi: 10.1128/mBio.01242-21 (PMC8406200; doi:10.1128/mBio.01242-21)
Supplement: FIG S4 [file mbio.01242-21-sf004.pdf]

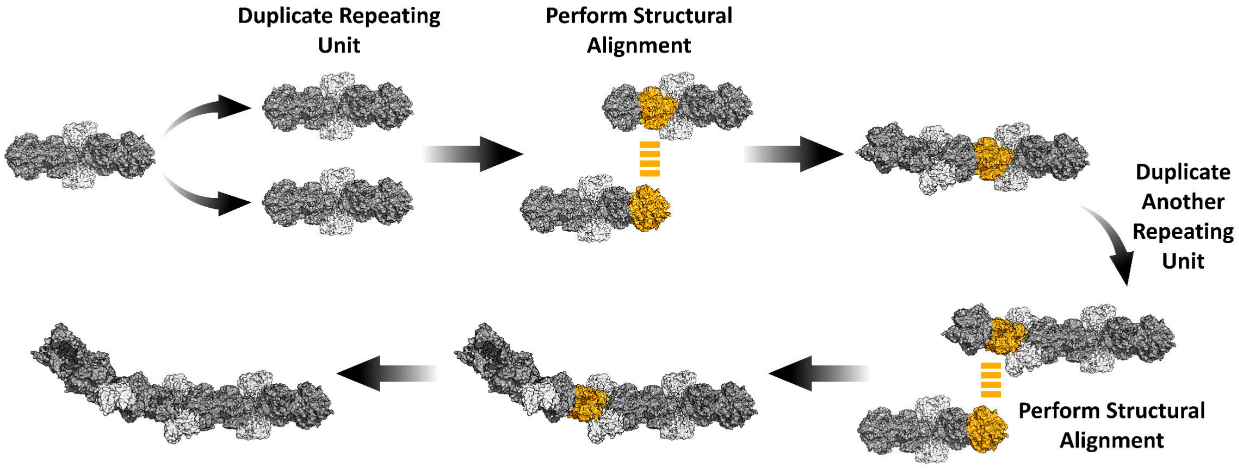
Duplicate Repeating Unit
Perform Structural Alignment
Duplicate Another Repeating Unit
Perform Structural Alignment
