# Supplementary material for: SAMase of Bacteriophage T3 Inactivates Escherichia coli’s Methionine S-Adenosyltransferase by Forming Heteropolymers
Source: mBio. 2021 Aug 3;12(4):e01242-21. doi: 10.1128/mBio.01242-21 (PMC8406200; doi:10.1128/mBio.01242-21)
Supplement: TABLE S1 [file mbio.01242-21-st001.pdf]

Table S1: Data collection and refinement parameters of the cryo-EM reconstruction in this study

Electron Microscope	FEI Tecnai F30 Polara
Voltage (kV)	300
Electron Detector	K2 summit
PDB ID/EMDB Codes	7OCK/12809
FSC(1/f)=0.143 Masked/Unmasked (Å)	3.6/3.9
Number of micrographs	538
Number of particles	65950
Total dose (e-/Å2)	80
Pixel size (Å)	1.1
frames/movie	50
