# Supplementary material for: SAMase of Bacteriophage T3 Inactivates Escherichia coli’s Methionine S-Adenosyltransferase by Forming Heteropolymers
Source: mBio. 2021 Aug 3;12(4):e01242-21. doi: 10.1128/mBio.01242-21 (PMC8406200; doi:10.1128/mBio.01242-21)

**A**

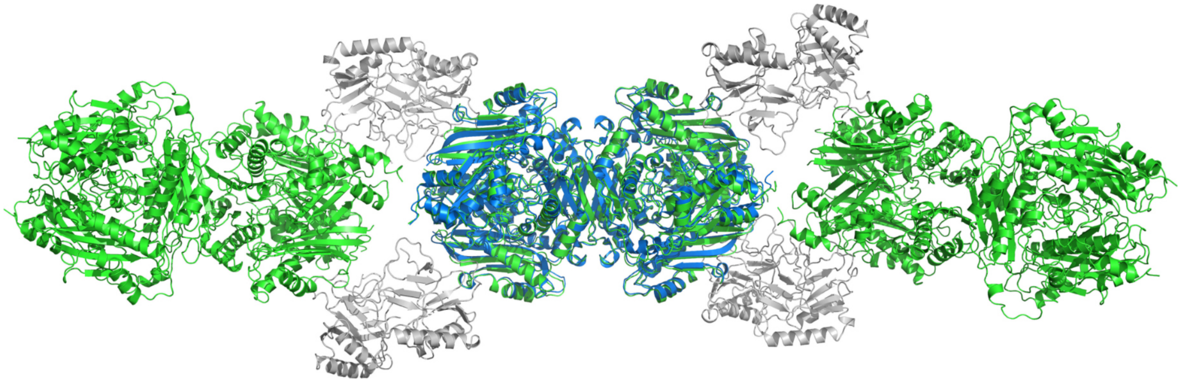

**B**

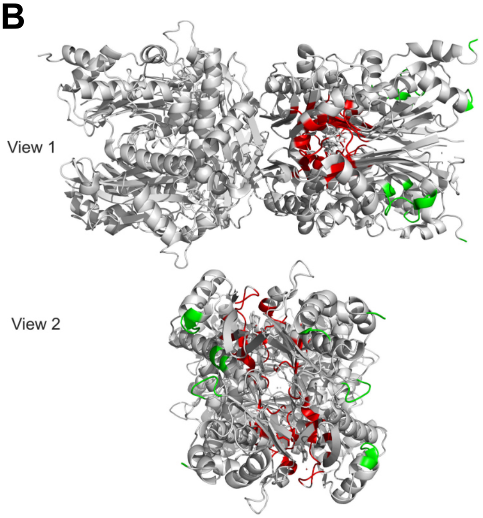


**Pocket residues**

| Axis index | 1 | 2 | 3 | 4-7 | 6-7 | 8-10 | 11-13 | 14 | 15 | 16 | 17 |
|---|---|---|---|---|---|---|---|---|---|---|---|
| Pocket residue (chain A) | 40A | 42E | 55E | 98Q-99S | 101D-102I | 117G-119Q | 259G-261A | 265K | 269K | 271D | 302I |
| Axis index | 18 | 19 | 20-22 | 23-25 | 26-27 | 28-30 | 31-37 | 38-40 | 41-42 | | |
| Pocket residue (chain B) | 8E | 10V | 14H-16D | 163D-165K | 186S-187T | 225N-227T | 228R-234G | 237G-239C | 244R-245K | | |

**Interface residues**

| Axis index | 1-5 | 6-12 | 13-14 | 15-17 | |
|---|---|---|---|---|---|
| interfacial residue (chain A) | 128N-132V | 208K-214E | 320V-321P | 382G-384K | |
| Axis index | 18-22 | 23 | 24-25 | 26-32 | 33-34 |
| interfacial residue (Chain B) | 128N-132V | 136A | 139T-140Y | 208K-214E | 383G-384K |

**C**

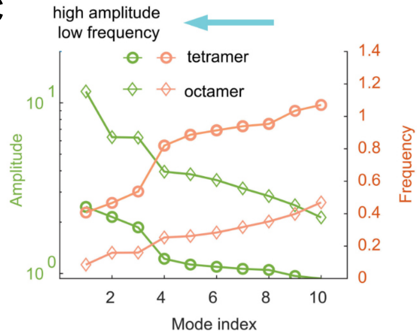


**D**

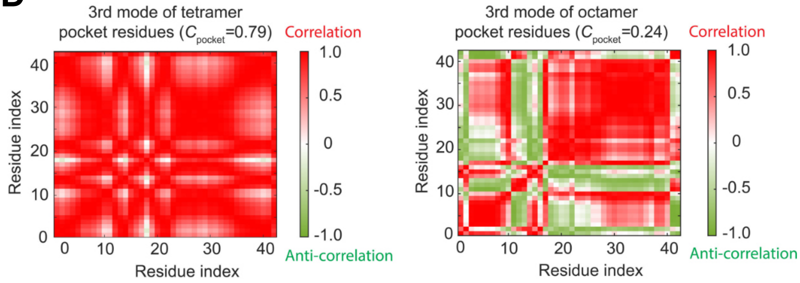


**E**

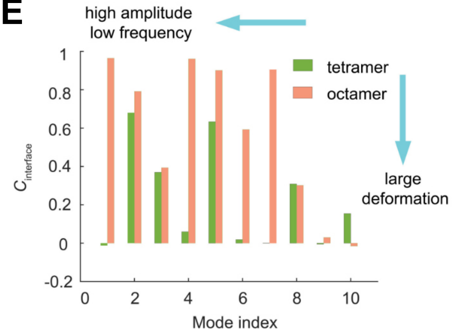


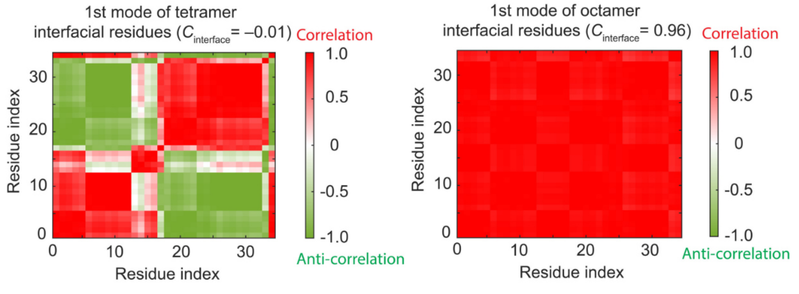

Supplement: FIG S8 [file mbio.01242-21-sf008.pdf]
